# Gradients in pore size enhance the osteogenic differentiation of human mesenchymal stromal cells in three-dimensional scaffolds

**DOI:** 10.1038/srep22898

**Published:** 2016-03-10

**Authors:** Andrea Di Luca, Barbara Ostrowska, Ivan Lorenzo-Moldero, Antonio Lepedda, Wojcech Swieszkowski, Clemens Van Blitterswijk, Lorenzo Moroni

**Affiliations:** 1 University of Twente, Tissue Regeneration Department, Drienerlolaan 5, 7522 NB, Enschede, The Nederlands; 2 Division of Materials Design, Faculty of Materials Science and Engineering, Warsaw University of Technology, 02-507, Warsaw, Poland; 3 Maastricht University, MERLN Institute for Technology Inspired Regenerative Medicine, Complex Tissue Regeneration department, P. Debyelaan 25, 6229 HX Maastricht, The Netherlands; 4 Universita’di Sassari, Department of Biomedical Sciences, Via Muroni 25, Sassari, Italy

## Abstract

Small fractures in bone tissue can heal by themselves, but in case of larger defects current therapies are not completely successful due to several drawbacks. A possible strategy relies on the combination of additive manufactured polymeric scaffolds and human mesenchymal stromal cells (hMSCs). The architecture of bone tissue is characterized by a structural gradient. Long bones display a structural gradient in the radial direction, while flat bones in the axial direction. Such gradient presents a variation in bone density from the cancellous bone to the cortical bone. Therefore, scaffolds presenting a gradient in porosity could be ideal candidates to improve bone tissue regeneration. In this study, we present a construct with a discrete gradient in pore size and characterize its ability to further support the osteogenic differentiation of hMSCs. Furthermore, we studied the behaviour of hMSCs within the different compartments of the gradient scaffolds, showing a correlation between osteogenic differentiation and ECM mineralization, and pore dimensions. Alkaline phosphatase activity and calcium content increased with increasing pore dimensions. Our results indicate that designing structural porosity gradients may be an appealing strategy to support gradual osteogenic differentiation of adult stem cells.

Regenerative medicine is a multidisciplinary field aiming to regenerate tissues by combining biological factors and engineering fundamentals^1^. Recently the use of stem cells in regenerative medicine has gained momentum thanks to their capacity to differentiate into multiple lineages^2^,^3^. Human mesenchymal stem/stromal cells (hMSCs) can undergo chondrogenic, osteogenic and adipogenic differentiation^3^, among others, and are not associated to the ethical concerns of other stem cells like embronic ones. hMSCs differentiation has been reported to depend on environmental cues such as oxygen and nutrient availability^4^,^5^, pore size^6^,^7^, material stiffness^8^, surface topography^6^,^9^ and more conventionally the adminisration of soluble factors^10^,^11^,^12^. All these parameters have been applied in the design and fabrication of scaffolds aiming at mimicking the natural three-dimensional (3D) environment where hMSCs reside.

Different processing techniques have been developed to build scaffolds for tissue engineered constructs^13^, among which solvent casting, salt or particulate leaching and gas foaming^14^. Although all these techniques are easy to implement, the resulting scaffolds present several drawbacks, such as the lack of completely interconnected pores, a limited control of the pore size and geometry, and the formation of tortuous pore networks associated to limited nutrient diffusion^15^,^16^. Conversely, additive manufacturing (AM) emerged in the past decade as an appealing tool to fabricate scaffolds with a controlled and completely interconnected pore network. This can be achieved thanks to the possibility to fine tune processing parameters such as fiber diameter, fiber spacing, layer thickness, and layer angle deposition. Additionally, the computer aided design/computer aided manufacturing (CAD/CAM) process governing AM technologies allows tailoring pore geometry and size in a layer-by-layer manner^17^,^18^. These parameters can be modulated in order to obtain a constant variation of the pore features within the same construct, thus forming structural gradients.

Gradients are present in the body leading a number of events and processes in the embryonic stage as well as in adult life. Structural gradients can be found in the body mainly at the interface between tissues. For example, processes such as osteochondral, tendon and ligament tissue development, as well as tumor formation, are governed by morphogens and oxygen gradients^19^,^20^,^21^. In the specific case of bone tissues, a structural gradient can be identified in a radial direction in long bones and in an axial direction in flat bones, presenting a variation in bone density from the cancellous bone to the coritical bone^22^. Clinically, current therapies for bone replacement, such as as autografts and allografts, are not yet completely successful, due to several drawbacks such as the donor-site morbidity, the limited tissue availability and surgery complications, highlighting that this procedures are not always a possible option^23^,^24^.

The concept of gradient has been applied in different studies in two dimensional (2D) systems to control or analyze cell differentiation^10^ and migration^25^,^26^. 3D scaffolds presenting a gradient structure could provide cues similar to the native enviroment and may guide stem cells to differentiate toward the lineage of the targeted tissue to be regenerated. In literature, several studies involving gradient scaffolds have been presened. In order to direct the differentiation of hMSCs in certain areas of the construct, gradients of growth factors^27^ and material stiffness^28^ were generated. To the best of our knowledge, no studies have linked the stem cell osteogenic differentiation with structural gradients in porosity and pore size. Besides improving cell seeding efficiency due to the higher number of fiber connections^29^, structural gradients can result in locally different concentrations of available nutrients. Therefore, we hypothesized that the creation of a gradient in scaffold porosity and pore size could influence hMSCs differentiation by impacting cell density and nutrient availability. Here, we fabricated 3D plotted scaffolds presenting an axial gradient in pore size and total porosity and assessed their effect on hMSCs osteogenic differentiation.

## Results

### Scaffold and gradient characterization

**Figure 1:**
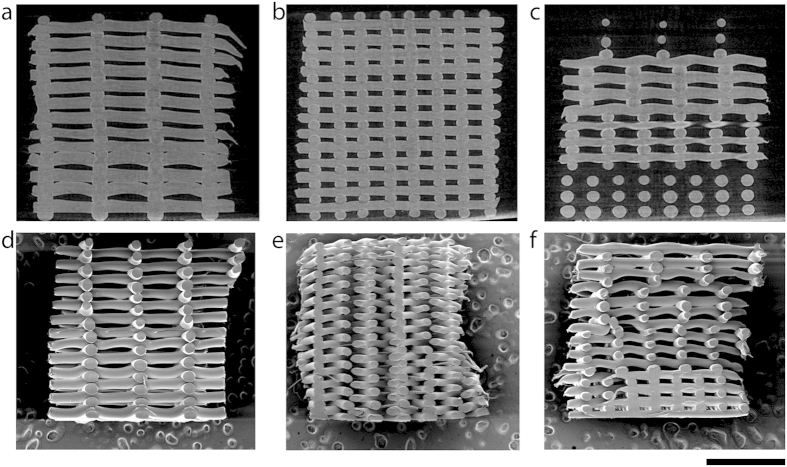
μCT and SEM micrographs displaying NG1100 (**a**,**d**), NG500 (**b**,**e**) and G scaffolds (**c**,**f**). Scale bar 2 mm.

Four zones in the gradient scaffolds can be distinguished (Fig. 1c,f), where the fiber spacing changed from bottom to top from 500 μm, to 700 μm, 900 μm, 1100 μm. Control scaffolds were printed by keeping the fiber spacing constant at 500 μm and 1100 μm (Fig. 1b,c,e,f). By increasing the fiber spacing, the volume of the pore in the different zones increased by 10 times from the smallest to the largest pore size. (Table S1). As expected, the overall porosity of the gradient scaffolds was in between the porosity of the controls (Table S2). The poroity of the different gradient areas increased from 58% ± 0.07% close to the porosity of the NG 500 (47.24% ± 6.9%), to 81% ± 0.04% that matched with porosity values of NG 1100 (80.63% ± 2.3%).

### hMSCs growth and Improved osteogenic differentiation in the gradient scaffolds

**Figure 2:**
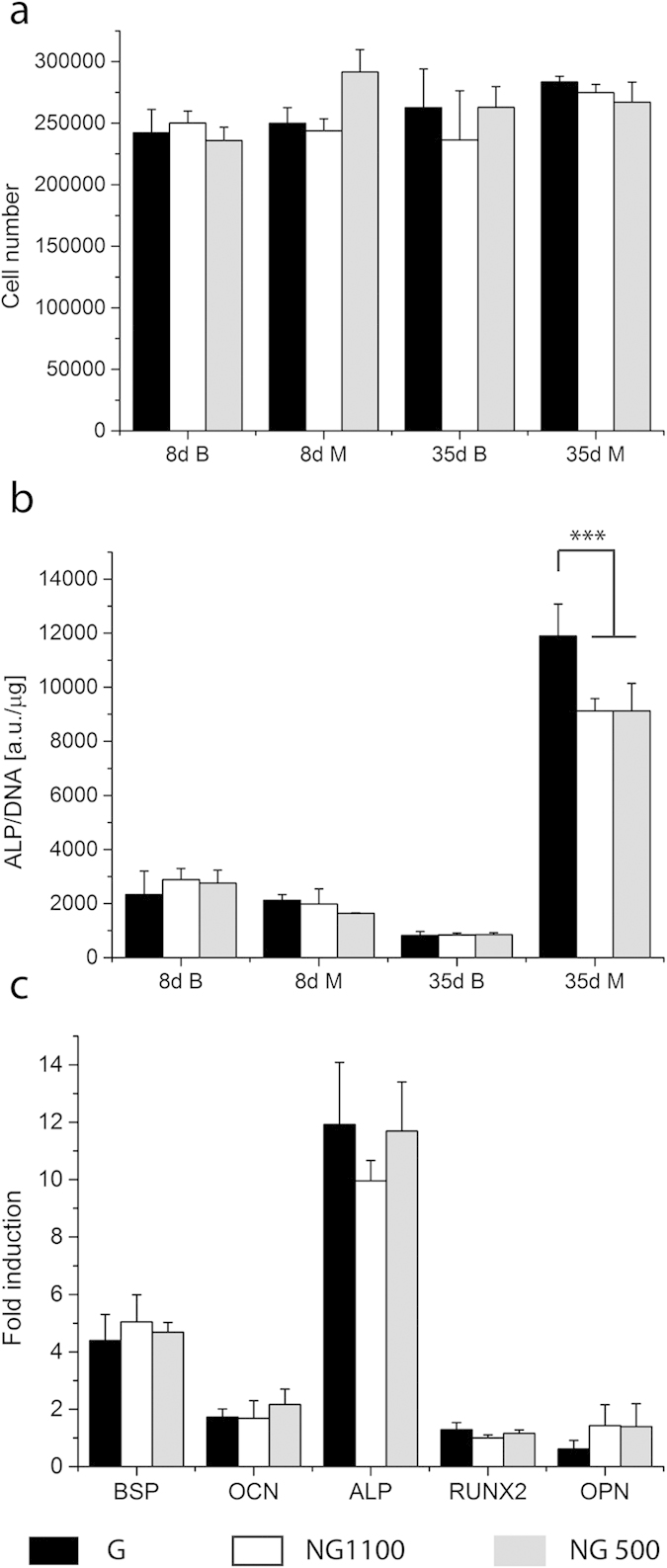
Cell number and ALP activity of D1 on 300PEOT55PBT45 normalized by μg of DNA after 8 and 35 days in culture (**a**,**b**) and fold induction of osteogenic markers after 35 days (**c**). After 1 day in differentiation media (8 days in culture) no differences in ALP activity or cell number were visible, whereas 4 weeks of differentiation enhanced ALP activity in gradient scaffolds with respect to the controls. The cell number remained similar in all conditions. BSP, OCN and ALP genes were upregulated, no major differences were shown among the gradient and non-gradient scaffolds. (***shows significant difference, p < 0.001, n = 3).

The amount of cells adhered on 300PEOT55PBT45 and PCL was around 325000 and 250000 cells per scaffold, respectively, which corresponded to a 65% cell seeding efficiency for 300PEOT55PBT45 and 50% cell seeding efficiency for PCL (Figures S1 and S2). Though not statistically different, 300PEOT55PBT45 seemed to perform better in terms of cell attachment. After 8 days from cell seeding, cell number on 300PEOT55PBT45 scaffolds remained constant around 250000 cells, without major differences among the conditions or type of construct. The overall cell number did not vary significantly at day 35, independently from the culture conditions or the type of construct (Fig. 2a), thus inferring that hMSCs did not proliferate significantly in these 3D scaffolds during the culture period.

hMSCs showed a basal ALP level (Fig. 2b). After 8 days of culture (7 days in proliferation medium and 1 day in basic or mineralization media), as expected, no main differences could be detected between the G and NG scaffolds cultured in basic and mineralization media. The basal ALP levels decreased in time in basic media in NG and G scaffolds. After 28 days in mineralization media the ALP levels markedly increased in all the samples. hMSCs cultured in G scaffolds showed significantly increased ALP activity levels with respect to the cells cultured in the NG scaffolds (Fig. 2b). As shown in Fig. 2c, after 28 days under mineralization conditions the expression of late osteogenic markers differentiation such as osteocalcin and osteopontin was not increased. Early markers such as bone sialoprotein (BSP) and. ALP were upregulated. BSP showed showed a 5-fold increase with respect to the cells cultured in basic medium within the NG 500 construct. No major differences were noticed among the gradient scaffolds and the controls. The expression of ALP was increased by 12 times. Yet, no significant differences could be seen.

**Figure 3:**
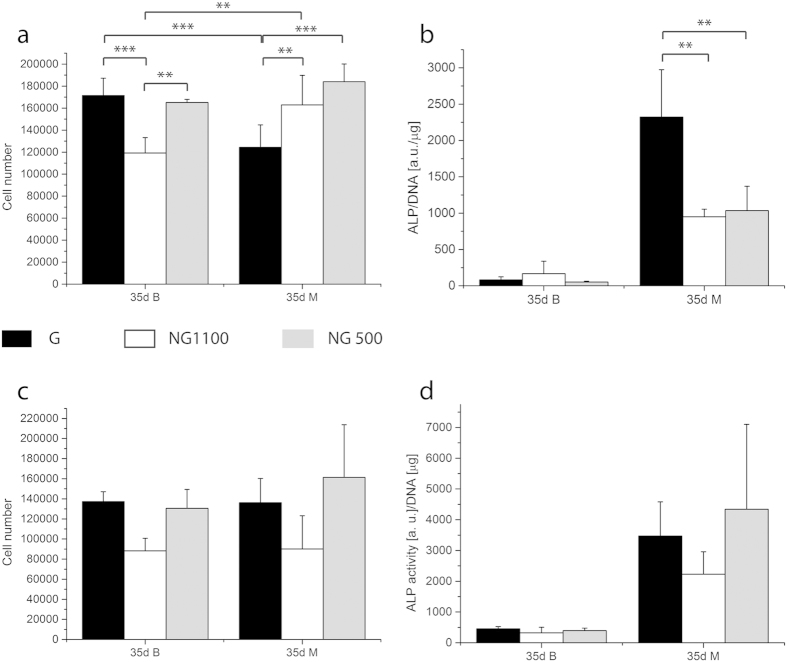
Cell number and ALP activity of donor 2 on 300PEOT55PBT45 scaffolds (**a**,**b**) and donor 1 on PCL scaffolds (**c**,**d**). Under basic conditions NG1100 scaffolds displayed a significantly lower cell number after 35 days of culture; under mineralization conditions the gradient showed a lower cell number. The ALP activity confirmed the results showed by donor 1 on 300PEOT55PBT45 scaffolds, the gradient significantly improved the ALP activity with respect to the non gradient scaffolds. When cultured on PCL scaffolds hMSCs didn’t display any significant differences in terms of cell number and ALP activity in basic as well as in mineralization medium. **shows statistical significance p < 0.01 and ***p < 0.001.

In order to confirm the results obtained in terms of osteogenic differentiation, another hMSCs donor was tested in G, NG 500 and NG 1100 scaffolds. As the main differences were seen after 28 days in differentiation condition only this time point was tested. Cell numbers as well as the ALP activity in all the constructs were lower compared to the first donor. Yet, ALP activity was significantly higher in G scaffolds than in the controls (Fig. 3b), thus confirming what observed for the first donor. If the results of the 2 donors are normalized by the NG 1100 or NG 500 values, the second donor displayed a higher induction factor with a 2.5 fold increase in G scaffolds compared to NG scaffolds. The first donor showed an increased ALP activity of 25% in G scaffolds compared to NG ones (Figure S3). Cell number under basic conditions was significantly lower in NG1100, whereas under mineralization conditions the NG scaffolds showed a higher cell number compared to the G scaffolds (Fig. 3a). In order to understand whether the scaffold material chemistry could influence the differentiation, the same analysis was conducted on scaffolds made of PCL. Despite lack of statistical significance, both cell number and ALP activity seemed to be higher in the control NG 500 compared to NG 1100. The gradient showed values that resembled the ones of the NG 500 control (Fig. 3c,d). In addition, osteocalcin production also increased in mineralization medium in G and NG 1100 scaffolds compared to NG 500 scaffolds (Figure S4).

### Pore size driven differentiation of hMSCs

**Figure 4:**
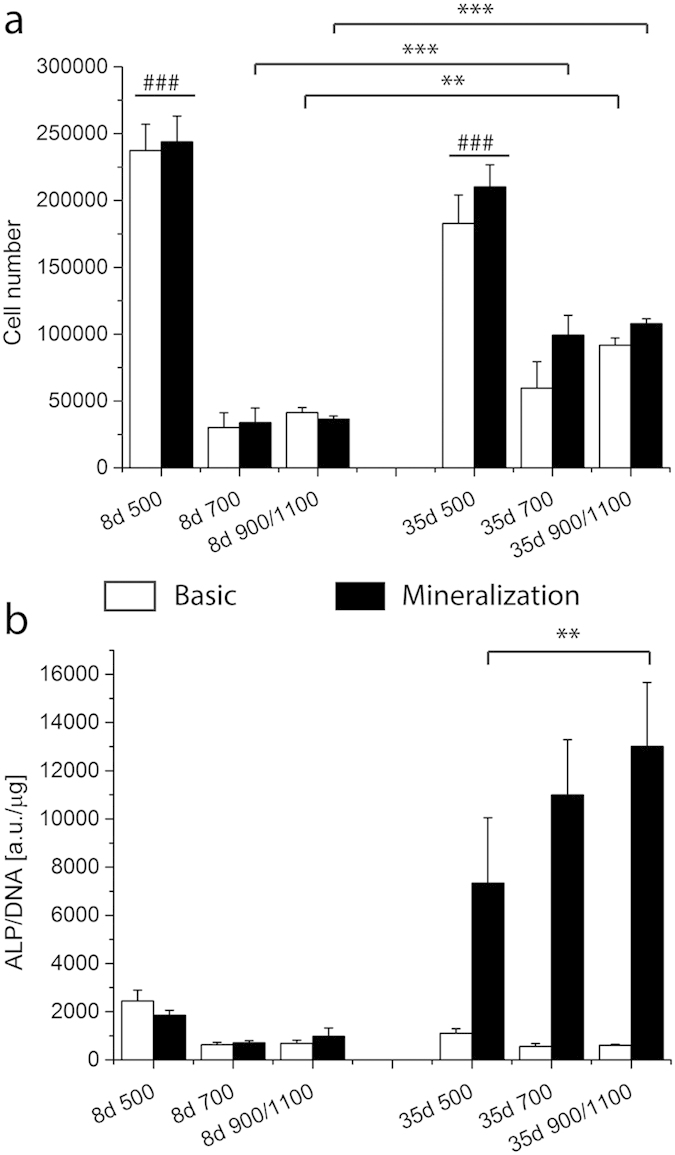
Cell number (**a**) and ALP activity (**b**) of D1 on 300PEOT55PBT45 are shown per gradient zone at 8 and 35 days. The cell number was significantly higher in the smallest pore size compared to the other areas no matter neither the time point nor the culture conditions. At 35 days of culture the cell number in the 700 and 900/1100 zones significantly increased with respect to the previous time point. The ALP activity at 35 days under mineralization conditions showed an increase trend opposite to the pore size. Cells residing in the 900/1100 zone displayed a significantly higher ALP activity with respect to the ones located in the smallest pore zone. ^###^indicates statistical significance within the same time point in graph a, p < 0.001; **, ***depict statistical significance within the same conditions p < 0.01 and p < 0.001 respectively.

As the overall ALP activity level increased in the G scaffolds compared to the controls, a partition analysis on the different areas of the gradient scaffolds was performed to better understand the differences among the porosity zones of the G scaffold. After 8 days cells were localized mainly in the zone with the lowest porosity independently from the media used (Fig. 4a). The ALP activity of cells localized in the small pore zone was higher with respect to the other areas after 1 day in basic and mineralization media (Fig. 4b). This trend was inverted after culturing in differentiation media. ALP activity levels of the cells cultured in mineralization medium increased over time in all the partitioned areas (Fig. 4b). The ALP activity increased with increasing the pore size.

**Figure 5:**
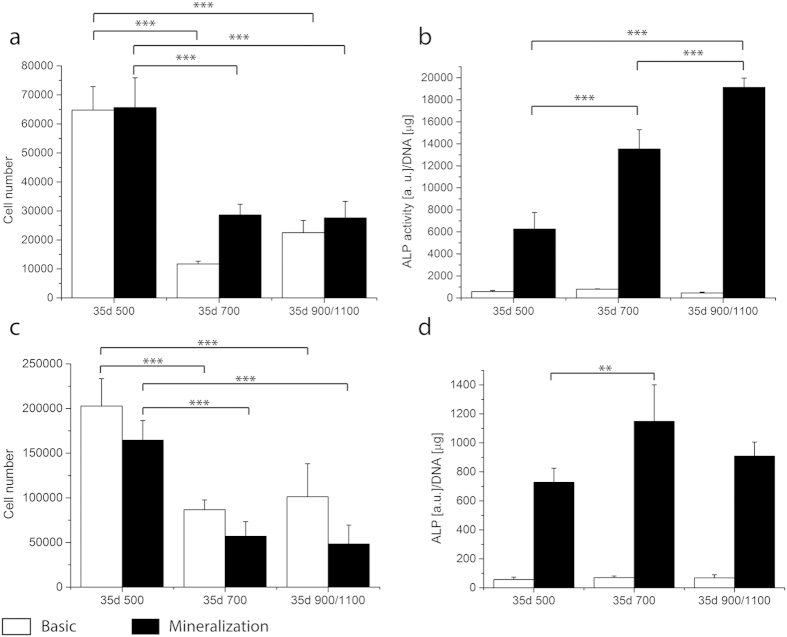
Cell number and ALP activity per gradient zone of donor 1 hMSCs cultured on PCL scaffolds (**a**,**b**) and donor 2 on 300PEOT55PBT45 scaffolds (**c**,**d**). After 35 days the highest cell number is located in the area with the smallest pores. ALP activity displayed the same trend when donor 1 was cultured on PCL scaffolds, and a similar trend for donor 2 on 300PEOT55PBT45 scaffolds, with the lowest level in the 500 zone and the highest in 700 and 900/1100. **and ***statistical significance p < 0.01 and p < 0.001.

Also for the partition analysis, the second donor and scaffolds in PCL were tested. After 35 days of culture in both conditions cells were located in higher number in the small pore zones for both donors and materials (Fig. 5a,c). Cells showed an increase in ALP activity with the increase in pore size for donor 1 in PCL (Fig. 5b). D2 in 300PEOT55PBT45 confirmed the lowest ALP levels where pores were small. No major differences could be seen between the 700 and 900/1100 zones (Fig. 5d). To further understand whether the observed gradual increase of ALP activity in the different regions of the G Scaffolds could be related to a different cell density, ALP activity was also normalized by cell number/pore volume. Results confirmed an increased ALP activity with increasing pore size (Figure S5).

### ECM and mineralization analysis

**Figure 6:**
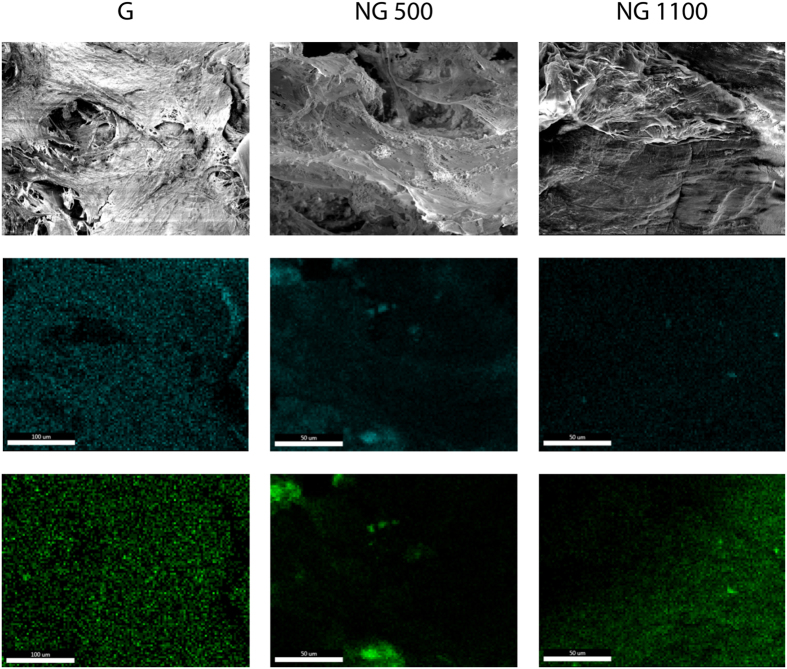
SEM micrograph and EDAX scan at day 35, revealing calcium (blue) and phosphate (green) for G and NG scaffolds. The co-localization of the colors suggested the presence of early ECM mineralization process. Scale bar 50 μm.

After 8 days in culture, pores were partially filled by ECM that looked fibrous in both culture conditions. After 35 days the amount of ECM increased and differentiated between the two conditions. Whereas in basic medium ECM kept having a dense fibrous appearance, in mineralization conditions some nodules were localized on top of the dense fibrillar ECM structure. In order to confirm the possible mineralization of the ECM in correspondence to the observed nodules, an EDAX analysis was performed. In all the samples the mapping showed a co-localization of the colors representing both the calcium signal and the phosphate signal. The intensity of the two elements signal increased in correspondence of mineralization nodules. Qualitatively, nodules looked bigger in G and NG500 scaffolds compared to those found in the ECM of the NG1100 scaffolds (Fig. 6).

**Figure 7:**
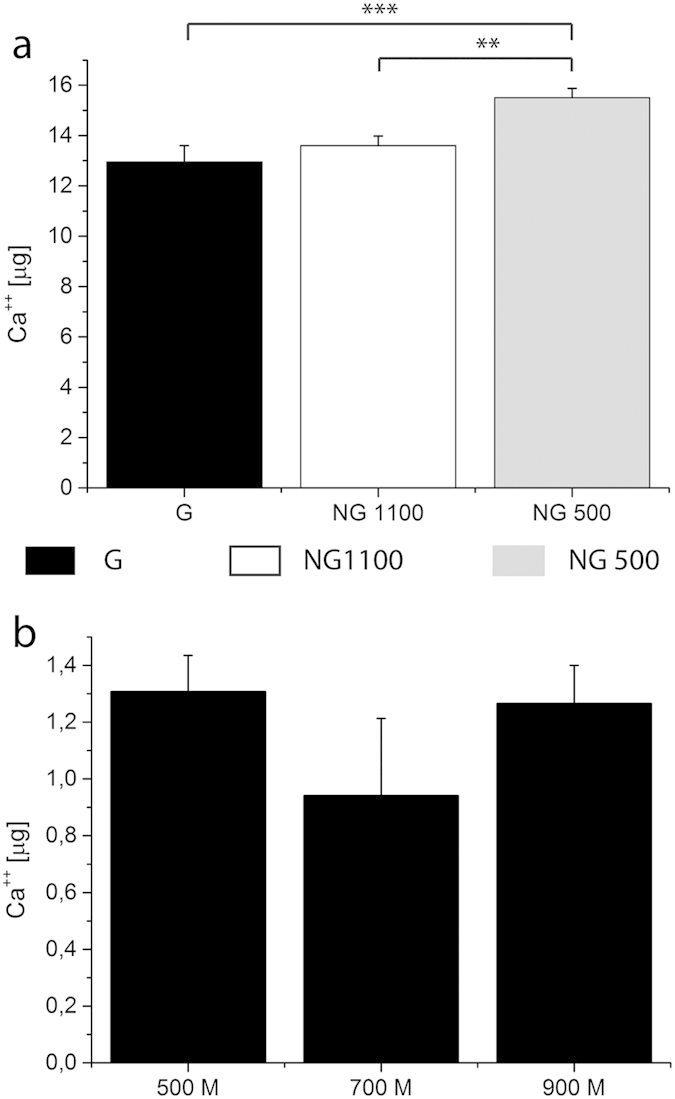
Calcium assay was performed on full scaffolds (**a**) as well as on partitioned gradient scaffolds (**b**) cultured under mineralization conditions for 28 days. Full scaffolds displayed an opposite trend in calcium content. The partition analysis did not show any significant difference among the gradient portions. **and ***statistical significance p < 0.01 and p < 0.001 respectively.

**Figure 8:**
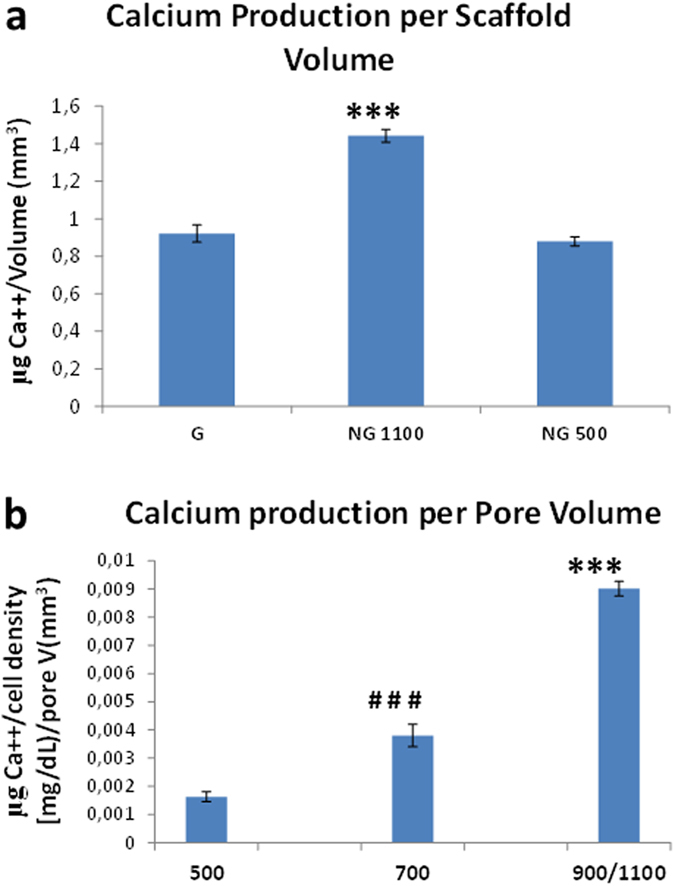
Calcium assay normalized by scaffold volume (**a**) as well as partition analysis of calcium levels normalized by cell density (**b**) cultured under mineralization conditions for 28 days. ***shows statistical significance p < 0.001 with respect to other scaffolds (**a**) or other regions. ^###^shows statistical significance p < 0.001 compared to other gradient scaffold regions.

In order to quantify and confirm whether there were differences in the mineralization levels in the constructs, a calcium assay was performed. It was possible to observe an inverse trend in calcium content with respect to the porosity. Calcium levels were significantly higher in NG 500 compared to G and NG1100 scaffolds (Fig. 7a). The partition analysis did not show any significant difference between the gradient zones (Fig. 7b). However, when the scaffold volume was considered, NG1100 showed the highest calcium levels in full scaffolds. Furthermore, when cell density was considered in the partition analysis, an increasing amount of calcium per cell density was measured with increasing pore size, thus confirming ALP results (Fig. 8). To further dissect the role of cell density variations with varying pore size, we reasoned that differential availability of nutrients would be present in the different regions of gradient scaffolds. In particular, oxygen levels could decrease more rapidly with decreasing the pore size, thus leading more easily to higher hypoxic regions^30^. To prove this hypothesis, we measured hypoxia inducible factor (HIF)-1α and HIF-2α. Results showed an increase of both HIFs with decreasing pore size (Figure S6).

## Discussion

Several strategies can be used to obtain a scaffold with a porosity gradient. Among them, fused deposition modeling emerged due to the possibility to fine tune several features such as fiber diameter, height and distance between the fibers. In the present work the fiber height (or layer thickness) was maintained constant and the fiber spacing was gradually increased along the z axis. Scaffolds were homogeneously fabricated. The fiber size could experience small variations in the case of G scaffolds due to the fact that with increasing the fiber spacing, and consequently the pore size, a larger distance has to be covered by the fibers before reaching the next contact point. This can create fiber size fluctuations due to the associated larger deformation of the molten polymer strand. Gradient scaffolds led to an axial gradient in pore size that affected the distribution and differentiation of hMSCs seeded in the resulting scaffolds. As already shown in previous studies, the design of the scaffolds and the pore geometry affect cell behavior in terms of adhesion, localization, ECM deposition and differentiation. Woodfield *et al.* proposed a 300PEOT55PBT45 gradient scaffolds for the zonal characterization of cartilage using primary chondrocytes^31^. More recently, Sobral *et al.* described cell adhesion and localization on gradient versus non gradient scaffolds, showing an improved cell seeding efficiency and distribution of an osteosarcoma cell line (SaOS) within the gradient scaffolds with respect to the non-gradient ones^29^. Despite what reported in literature, cell adhesion on the gradient and non-gradient scaffolds didn’t show any significant difference in our study. This might be due to the different cell source used in our study, as it is known that hMSCs are more sensitive to environmental changes than already differentiated cells and cell lines. A difference between 300PEOT55PBT45 and PCL was observed, being the first one more favorable for cell adhesion, which might be due to a higher hydrophilicity (Figure S1).

ALP is an enzyme responsible for the dephosphorylation of several molecules including nucleotides and proteins. ALP has always been related to osteoblast differentiation, as an increase of the enzyme activity was observed in the early stages of their commitment^32^. ALP activity in the gradient scaffolds was significantly higher after 28 days in mineralization medium in both donors compared to the non-gradient scaffolds. Despite the different levels of intensity, both donors showed a significantly higher ALP activity compared to the control in 300PEOT55PBT45 scaffolds. When presented as fold induction (Figure S3), the gradient increased ALP activity of the first donor by 25% and for the second donor by 2.5 times. The fold increase did not change when either NG500 or NG1100 were chosen as baseline. In case of PCL scaffolds, we could not observe a statistical difference between G and NG scaffolds at a full scaffold level, despite higher ALP values for NG500 and G scaffolds (Fig. 3d). This could be associated to the fact that PCL was shown to preferentially support chondrogenic more than osteogenic differentiation^33^. The partition analysis further highlighted the effect of the porosity on hMSCs differentiation when they were cultured within the same constructs for both polymer chemistries, thus showing that also PCL supports a differential expression of ALP activity, which increased with increasing the pore size (Figs 4b and 5, and Figure S4 in supporting information). Therefore, further studies on additional biomaterial chemistries should be performed to conclude whether the influence of pore size gradients on hMSCs osteogenic differentiation is specific for 300PEOT55PBT45 only or a more general phenomenon common to other biomaterials.

The cell number was significantly higher in the small pore zone at 8 and 35 days under basic and differentiation conditions. Comparing the cell number at 8 and 35 days it decreased in the 500 μm zone and increased in the 700 μm and 900/1100 μm zones. Since from the full analysis of the fabricated scaffolds it was shown that the cell number per scaffolds remained constant, a migration from the small pores toward the bigger ones can be hypothesized. Probably due to lower nutrient available determined by the higher number of cells and by the small pore size, hMSCs were stimulated to migrate toward pores with lower cell density and higher nutrient and oxygen availability^34^,^35^.The re-distribution of cells along a gradient in oxygen tension was shown by Ardakani *et al.*^36^, thus corroborating our hypothesis. ALP activity showed an opposite trend with respect to the cell number. Although after 1 day in differentiation media (8 days in culture) no significant differences were seen between the areas of the gradient, after 28 days in differentiation media a gradient in ALP activity was generated having its maximum in the zone with the largest pores. Hsu *et al.* recently proved that under hypoxia condition hMSCs differentiation toward the osteogenic lineage is impaired and the level of ALP expression attenuated^30^. A possible explanation could be found in the differences in pore size. Smaller pores closed faster, determining a longer lack of nutrient and oxygen availability for the cells residing in that zone with respect to the cells localized in the bigger pores areas. Thanks to the pore size change, the oxygen local concentration could have changed in the different areas of the G scaffolds. Therefore, more than cell density, oxygen distribution or pore size alone, these three factors seemed to be connected as a pore size increase resulted in a lower cell density, which consequently increased the local oxygen and nutrient availability. This could explain why the trend in ALP activity followed the direction of the gradient, which was further corroborated by a correspondent decrease of HIF-1α and HIF-2α with increasing pore size. The molecular mechanism behind this phenomenon is still unclear and should be subject of further studies.

At the genetic level there were no major differences after 28 days of culture in relation to the influence of the analyzed structural scaffold gradient on hMSCs differentiation. The increased expression of early genes such as ALP and BSP and, on the other hand, the missed upregulation of late markers such as OCN and OPN might suggest that the culture of hMSCs on these polymeric scaffolds didn’t lead to a complete differentiation up to the later stages of mature osteoblasts. Despite the matrix mineralization followed a different trend compared to the ALP when looking at absolute calcium content, being the highest in the NG500 and the lowest in the NG1100 scaffolds, the same increasing trend with increasing pore size found for ALP was observed when taking into consideration cell density and pore volume.

Since the seeding procedure was performed by keeping the largest pores on top and the scaffold direction was not changed during culture, the gradient structure determined a higher localization of cells in the area with the small pores. To confirm that the seeding direction could play a role in the localization of the cells, the scaffolds were seeded and cultured in both ways. The dispersion of the cells within the scaffolds seemed to be influenced by its position at the moment of the seeding, since higher cell numbers were found in the area located at the bottom at the moment of the seeding (Figure S2). By comparing the two seeding direction, the ones presenting the highest cell number was still the one with the smallest pore size at the bottom. This area was the one presenting the highest surface available for cell attachment and growth. During culturing the cell number slightly decreased in the small pores zone and increased in the other zones (Fig. 3b). This can be explained by 2 factors: i) the cells in the first week from the cell seeding were localized mainly in the area with the smaller pore size and evenly distributed in the remaining 3 zones; in the following 4 weeks, they expanded in number mainly in the bigger pore areas; ii) in the area with the smallest pore size, pore closure due to ECM deposition was faster. This might have affected the diffusion of oxygen and nutrients including the soluble factors present in the media. On the other hand, with increasing pore size cells have more room to grow and deposit the matrix. Filling of the pores with formed ECM took longer, ensuring a longer and better nutrient and oxygen diffusion. Our results showed an initial differentiation of hMSCs toward the osteogenic lineage. To clarify whether polymeric scaffolds are able to support a full differentiation *in vitro*, a longer study can be foreseen. Additionally, an animal study should be performed in order to define the effect of pore size in tissue formation in an ectopic location like a subcutaneous or intramuscular implantation. A further step should be the implantation of the gradient and non-gradient scaffolds in an orthotopic defect in a large animal models in order to study their performance in the healing of a critical defect.

## Conclusion

A scaffold displaying a gradient in pore size along the Z axis was plotted by varying the fiber spacing and its influence on hMSCs differentiation was evaluated. The differentiation toward the osteogenic lineage of hMSCs cultured in mineralization media was improved in gradient scaffolds. The biomaterial used to fabricate the scaffolds did not seem to play a role, since PCL and 300PEOT55PBT45 showed the same trend when analyzing the local ALP activity in the different pore size gradient regions. Cells residing in different areas of the gradient displayed a trend in osteogenic differentiation following the pore size, probably due to a better supply of nutrient and oxygen in the compartments with the largest pores. Taken together the findings of this study introduce pore size gradients as a structural factor that could be taken into consideration when combining scaffolds and hMSCs for bone tissue engineering purposes.

## Materials and Methods

### Scaffolds preparation

Scaffolds were fabricated via rapid prototyping (Bioscaffolder, SysENG, Germany). Scaffolds made of poly(ethylene oxide therephtalate)/poly(butylene therephtalate) (PEOT/PBT) and of poly(ε-caprolactone) (PCL) were produced. PEOT/PBT is a family of block co-polymers characterized by an *a*PEOT*b*PBT*c* nomenclature, where *a* is the molecular weight of the starting PEG block and *b* and *c* are the PEOT/PBT ratio. Scaffolds made of both PCL and 300PEOT55PBT45 were already used in surgery and clinical trials are currently ongoing^37^. Briefly the polymers were placed in a stainless syringe and processed at 200 °C (300PEOT55PBT45, PolyVation, The Netherlands) and 100 °C (PCL, Sigma-Aldrich, USA). The molten polymer was extruded through a cartridge unit, by the application of a nitrogen flow with a pressure of 5 bar from a pressurized cap and an extrusion screw rotation of 200 rpm.

During plotting, the needle diameter, layer thickness and speed were kept constant at 200 μm, 150 μm and 180 mm/min, respectively. The fiber spacing was kept constant to 500 μm and 1100 μm for non-gradient (NG) scaffolds and varied from 500 μm to 1100 μm for gradient (G) scaffolds. The fiber spacing was changed every millimeter. The scaffolds were plotted in blocks of 20 × 20 mm and 4 mm in height. The tested samples were 4 × 4 mm cylinders punched out from the blocks.

### Cell expansion and culture

Human mesenchymal stromal cells (hMSCs) were isolated from the bone marrow of donors with written informed consent^38^. Aspirates were resuspended using a 20G needle and plated at a density of 0.5 million mono-nucleated cells per cm^2^. Cells were grown in MSC proliferation medium, which contains minimal essential medium (α-MEM, Gibco, Breda the Netherlands) supplemented with 10% fetal bovine serum (FBS, Lonza), 100 U/ml penicillin (Gibco, breda the netherlands), 10 μg/ml streptomycin (Gibco, USA), 2 mM L-glutamin (Gibco, breda the netherlands), 0.2 mM L-ascorbic acid 2-phosphate magnesium salt (ASAp, Sigma-Aldrich, Zwijndrecht, The Netherlands) and 1 ng/ml of basic fibroblast growth factor-2 (bFGF-2, Fisher Scientific, Landsmeer, the Netherlands) at 37 °C in a humid atmosphere with 5% CO_2_. Cells were expanded up to approximately 80% confluency and either frozen for further use or seeded on the scaffolds.

### Cell seeding on scaffolds

Two different hMSCs donors were used in this study. D249 (D1, age 72, female) and D8004L (D2, age 22, male). For donor D249, bone marrow aspirates were obtained and experiments approved by the Medical Ethical committee of the local hospital (Dutch: Medisch Ethische Toetsingscommissie (MECT) van het Medisch Spectrum Twente) following the Dutch national ethics guidelines from patients who had given written informed consent. For donor D8004L, pre-selected hMSCs were retrieved from the Institute of Regenerative Medicine (Temple, Texas), which has supplied standardized preparations of MSCs to hundreds of laboratories under the auspices of an NIH/NCRR grant (P40 RR 17447-06). Human bone marrow aspirates were obtained under a protocol approved by an institutional review board. Briefly, a bone marrow aspirate was drawn and mononuclear cells were separated using density centrifugation.

After trypsinization with 0.25% trypsin (Life Technologies, Bleiswijk, the Netherlands), cells (passage 2–4) were counted using a Bückner chamber and re-suspended in proliferation media at a density of 500000 cells in 40 μL. The day before seeding, scaffolds were disinfected in 70% EtOH for 30 min under stirring, washed 3 times in phosphate buffered saline solution (PBS) (Lonza, Breda, the Netherlands), and incubated overnight in cell proliferation media to allow protein adsorption on the scaffold’s fibers. After protein adsorption, the 40 μL of cell suspension were placed on the scaffolds in a drop wise fashion to account for a cell seeding density of 500000 cells/scaffold. The seeded scaffolds were placed for 4 hours in the incubator to allow cell adhesion before adding the cell culture medium.

Cells were cultured on the G and NG scaffolds for 7 days in proliferation media. At day 7, the proliferation media was changed and the cells within the scaffolds were cultured for another 1 and 28 days in basic medium, being the same as proliferation medium without bFGF, and mineralization medium, consisting of basic medium supplemented with 10 nM dexamethasone (Sigma-Aldrich, Zwijndrecht, The Netherlands) and 10 mM β-glycerol-phosphate (Sigma-Aldrich, Zwijndrecht, The Netherlands).

## Biochemical study

### DNA analysis

The cell number per scaffold was calculated from the μg of DNA, obtained by a Cyquant DNA assay kit (Life Technologies, Bleiswijk, the Netherlands). Briefly, each scaffold was cut to improve lysis efficiency and freeze-thawed 5 times. After the freeze-thawing process, cells within the scaffolds were lysated by diluting the 20x lysis buffer provided with the kit using a saline buffer (180 mM NaCl, 1 mM EDTA in distilled water). After 1 h of lysis, samples were sonicated 2 times for 10 seconds using a Branson sonifier 250 (Emerson Industrial Automation, USA). DNA content was quantified with a CyQuant kit (Invitrogen, Breda, the Netherlands) according to manufacturer’s protocol and fluorescence was measured at 480 nm using a spectrophotometer LS50B (Perkin Elmer, The Netherlands). DNA concentrations were calculated from a λ DNA standard curve.

### ALP activity

To evaluate hMSCs differentiation toward the osteogenic lineage, ALP content was measured using a CDP star kit (Roche, Woerden, The Netherlands). For this purpose, 10 μL of sample was added to a well of a white 96-well plate and 40 μL of substrate (Disodium 2-chloro-5-(4-methoxyspiro {1,2-dioxetane-3,2′-(5′-chloro)tricycle[3.3.1.13.7]decan}-4-yl)-1-phenyl phosphate) was added. After 15 minutes incubation, luminescence was read using a spectrophotometer LS50B (Perkin Elmer). ALP activity was corrected for DNA content.

### Osteocalcin quantification

Osteocalcin (OCN) production was analyzed after 35 days of culture (7 days in basic medium followed by 28 days in mineralization medium) using ELISA (human osteocalcin ELISA kit, Invitrogen). The test was performed on 5 samples per condition. Briefly, the medium was removed and samples were washed using ice cold PBS. A buffer was prepared using 890 volumes of miliQ water, 100 volumes of RIPA buffer (Cell Technologies) and 10 volumes of Halt™ protease and phosphatase inhibitor (Thermo Scientific). 150 microliters of the buffer was added to each samples, which were incubated on ice for 10 minutes. The buffer was then collected from the samples and centrifuged at 11000 g for 15 minutes. The supernatant was collected and used for the ELISA tests which were performed according to the manufacturer’s protocol. Briefly, 25 μl of samples and standard solutions were added to the osteocalcin antibody-coated strip- well plates. 100 μl of Anti-OST-HRP conjugate, respectively, were added to each correspondent well, and the plates were covered and incubated for 2 hours at room temperature. After incubation, the solutions in the wells were aspirated and the wells were washed 3 times using a washing solution provided in the kit. Then, 100 μl of a chromogen solution (Tetramethylbenzidine) was added to each well and the plates were incubated for 30 minutes at room temperature in the dark. Finally, 100 μl of stop solution was added to each well. The optical density of each well was read at 450 nm using a plate reader (MULTISKAN GO, Thermo Scientific). A standard curve was plotted in Microsoft excel and the concentration of OCN was determined in each well according to the standard curve.

### Gene expression analysis

For gene expression analysis the scaffolds were taken from the medium, washed twice with PBS, cut into small pieces and placed in an Eppendorf containing 750 μL of TRIzol® (Invitrogen) and stored at −80 °C. In the case of partition analysis the gradient scaffolds were cut in order to separate the gradient zones and the 3 samples were located in the same vial prior the addition of the TRIzol®, in order to ensure the collection of enough RNA. RNA isolation was performed by using a Bioke RNA II nucleospin RNA isolation kit (Bioke, Leiden, The Netherlands). 150 μL of CHCl_3_ were added and the vials were vigorously mixed, followed by a centrifugation at 12000 g for 15 minutes at 4 °C. The aqueous phase was transferred into a new tube and an equal amount of 70% ethanol was added. The mixture was transferred into a filter columns from the kit and the extraction was carried on by following the manufacturer’s protocol. RNA concentration and purity was evaluated via an ND1000 spectrophotometer (Nanodrop Technologies, USA); cDNA was synthetized using iScript™ (BIO-RAD, Veenendaal, The Netherlands) according to manufacturer’s protocol. Quantitative polymerase chain reaction (qPCR) was performed on the obtained cDNA by using the iQ SYBR®Gree Supermix (BIO-RAD, Veenendaal, The Netherlands) and the primers listed in Table 1. PCR reaction was carried out on the MyiQ2 Two-Color Real-Time PCR Detection System (BIO-RAD, Veenendaal, The Netherlands) under the following conditions, the cDNA was denatured for 10 minutes at 95 °C, followed by 45 cycles, consisting of 15 seconds at 95 °C, 15 seconds at 60 °C and 15 seconds at 72 °C. A melting curve was generated from each reaction to test the presence of primer dimers and aspecific products. The cycle threshold was calculated by the Bio-Rad iQ5 optical system software, in which the threshold was set in the lower log-linear region of the fluorescent signal. Ct values were normalized by the B2M housekeeping gene and ΔCt ((average of Ct control)-Ct value). Results were expressed as fold induction in mRNA expression normalized to the gene expression of the NG500 control scaffolds cultured in basic medium.

### Histological, computed tomography and SEM analysis

G and NG scaffolds were analyzed by scanning electron microscopy (SEM, Philips – XL 30 ESEM-FEG). Directly after plotting scaffold were punched, cut in half, gold sputtered and analyzed. SEM images were analyzed using Image J software in order to measure the fiber diameter, fiber spacing, and pore dimensions. Directly after plotting, scaffolds were also analyzed by computed tomography analysis using a source voltage and current of 40 kV and 250 μA respectively, 600 projections with an exposure of 100 ms and image pixel size of 3.97 μm.

Scaffolds cultured in mineralization medium for 28 days were fixed using 10% formalin, dehydrated by an increased series of ethanol concentration (50–60–70–80–90–96–100%) and cut in half. The final dehydration step was carried out using a Balzers CPD 030 Critical Point Drier.

Dry scaffolds were mounted on SEM stubs, gold sputtered (Cressington sputter coater 108 auto), and analyzed by Energy Dispersive X-Ray Analysis (EDAX, Ametek, USA). The picture of the area and the mapping of the localization of Calcium (Ca) and phosphate (P) element were acquired.

### Calcium assay

After 28 days in mineralization medium the scaffolds were collected, cut, sonicated and incubated in 1 M HCl solution for 3 days under agitation in order to release the Calcium. A QuantiChrom Calcium assay kit (DICA-500) (BioAssay System) was used to measure the calcium levels in each sample. The analysis was performed following the supplier protocol. Briefly, reagent A and B (provided with the kit) were mixed by combining equal volumes in order to obtain the working reagent. Five μL of each sample were pipetted in a clear 96 well plate and incubated for 3 minutes with 200 μL of working reagent. Optical density was read at 612 nm using a Multiscan Go (Thermo Scientific, Breda, The Netherlands) plate reader.

### Partition analysis

In order to analyze the behavior of hMSCs with the different pore sizes, at the end of the culture the gradient scaffolds were cut and the biochemical analysis was carried on the gradient zones separately. Due to the lower fiber number and the difficulty of separate them the areas with the biggest pores (900/1100) were analyzed together.

### Statistical analysis

All the quantitative data are expressed as mean ± standard deviation. Statistics were performed using IBM SPSS Statistics 20. A two-way ANOVA with Tukey as post-hoc test were used. Differences between experimental groups were considered significant when p ≤ 0.05.

## Additional Information

**How to cite this article**: Di Luca, A. *et al.* Gradients in pore size enhance the osteogenic differentiation of human mesenchymal stromal cells in three-dimensional scaffolds. *Sci. Rep.* **6**, 22898; doi: 10.1038/srep22898 (2016).

## Supplementary Material

Supplementary Information

## Figures and Tables

**Table 1:** Osteogenic markers used for the qPCR expressed as forward and reverse primer.

Gene	Forward Primer	Reverse Primer
B2M	ACAAAGTCACATGGTTCACA	GACTTGTCTTTCAGCAAGGA
ALP	ACAAGCACTCCCACTTCATC	TTCAGCTCGTACTGCATGTC
Runx2	TGGTTACTGTCATGGCGGGTA	TCTCAGATCGTTGAACCTTGCTA
Osteocalcin	TGAGAGCCCTCACACTCCTC	CGCCTGGGTCTCTTCACTAC
BSP	CCCCACCTTTTGGGAAAACCA	TCCCCGTTCTCACTTTCATAGAT
OPN	CTCCATTGACTCGAACGACTC	CAGGTCTGCGAAACTTCTTAGAT
