# Supplementary material for: Gradients in pore size enhance the osteogenic differentiation of human mesenchymal stromal cells in three-dimensional scaffolds
Source: Sci Rep. 2016 Mar 10;6:22898. doi: 10.1038/srep22898 (PMC4790631; doi:10.1038/srep22898)
Supplement: Supplementary Information [file srep22898-s1.pdf]

SUPPORTING INFORMATION

Gradients in pore size enhance the osteogenic differentiation of human mesenchymal stromal cells in three-dimensional scaffolds

Andrea Di Luca[1], Barbara Ostrowska[2], Ivan Lorenzo-Moldero[3], Antonio Lepedda[4], Wojcech Swieszkowski[2], Clemens Van Blitterswijk[1,3], Lorenzo Moroni[1,3]

[1]*University of Twente,Tissue Regeneration Department, Drienerlolaan 5,7522 NB, Enschede, The Nederlands;*

E-mail: a.diluca@utwente.nl

[2]*Division of Materials Design, Faculty of Materials Science and Engineering, Warsaw University of Technology, 02-507, Warsaw, Poland.*

[3] *Maastricht University, MERLN Institute for Technology Inspired Regenerative Medicine, Complex Tissue Regeneration department, P. Debyelaan 25, 6229 HX Maastricht, The Netherlands*

[4] *Universita'di Sassari, Department of Biomedical Sciences, Via Muroni 25, Sassari, Italy.*

Fiber spacing [µm]	Porosity [%]	Pore size [µm]	Pore volume [mm^3]
500	58%	270 ± 10	0.015 ± 0.002
700	70%	500 ± 30	0.055 ± 0.008
900	77%	710 ± 20	0.098 ± 0.009
1100	81%	870 ± 50	0.181 ± 0.026

Table S1. Table displaying the pore volume per gradient zone. The average volume increased by 10 times from the smallest to the largest pore size area.

Sample	Porosity [%]
G	67.83 ± 3.3
NG 500	47.24 ± 6.9
NG 1100	80.63 ± 2.3

Table S2. Table displaying the porosity of the full scaffolds. As expected the porosity of the G scaffolds were in between the porosity of the controls.

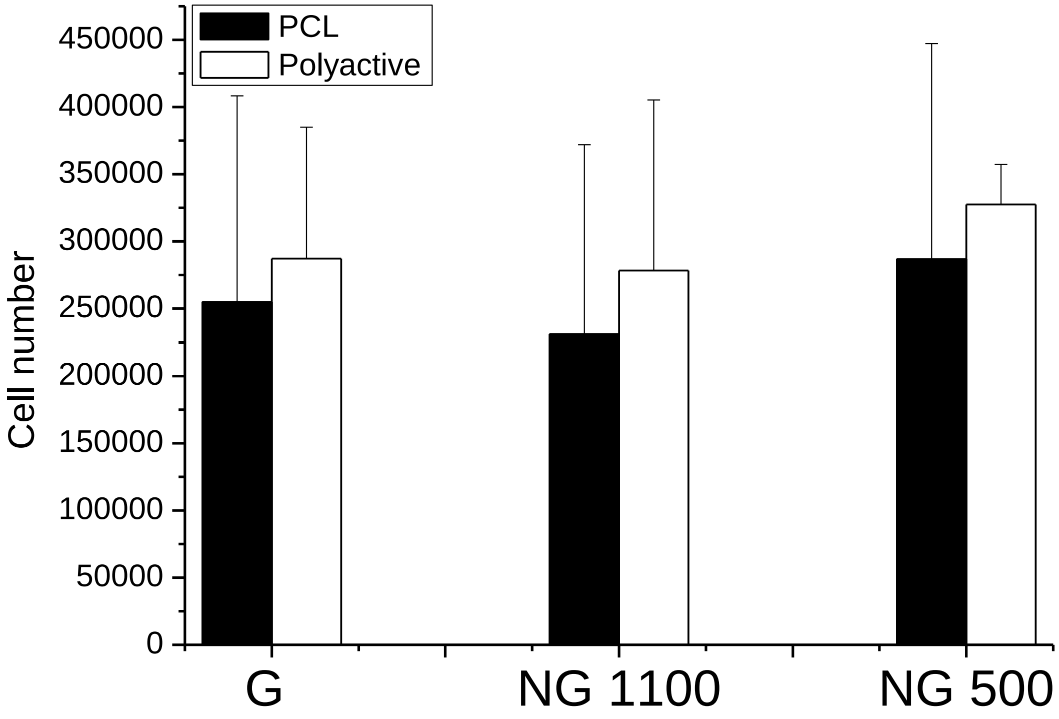

Figure S1. Cell seeding efficiency of 300PEOT55PBT45 after 8 hours. The two materials showed a similar cell seeding efficiency.

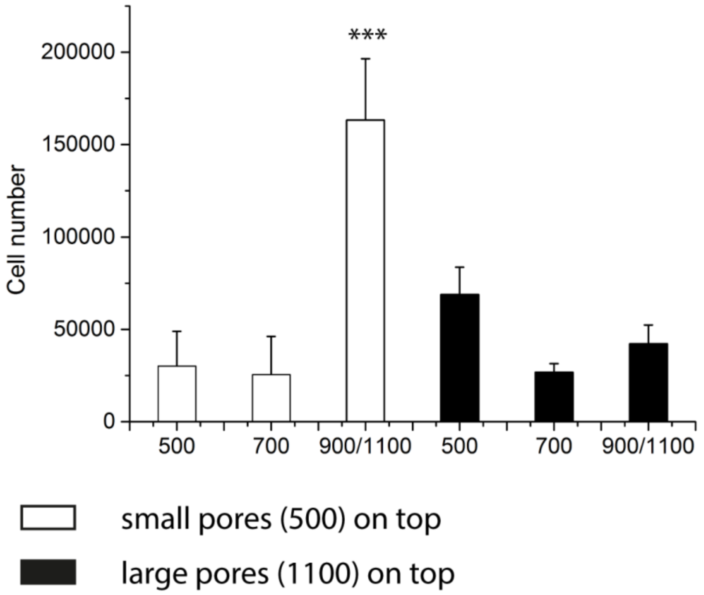

Figure S2. Plot showing the cell number per gradient zone related to the seeding direction. The highest number of cells is located in the area that at the seeding moment is located at the bottom.

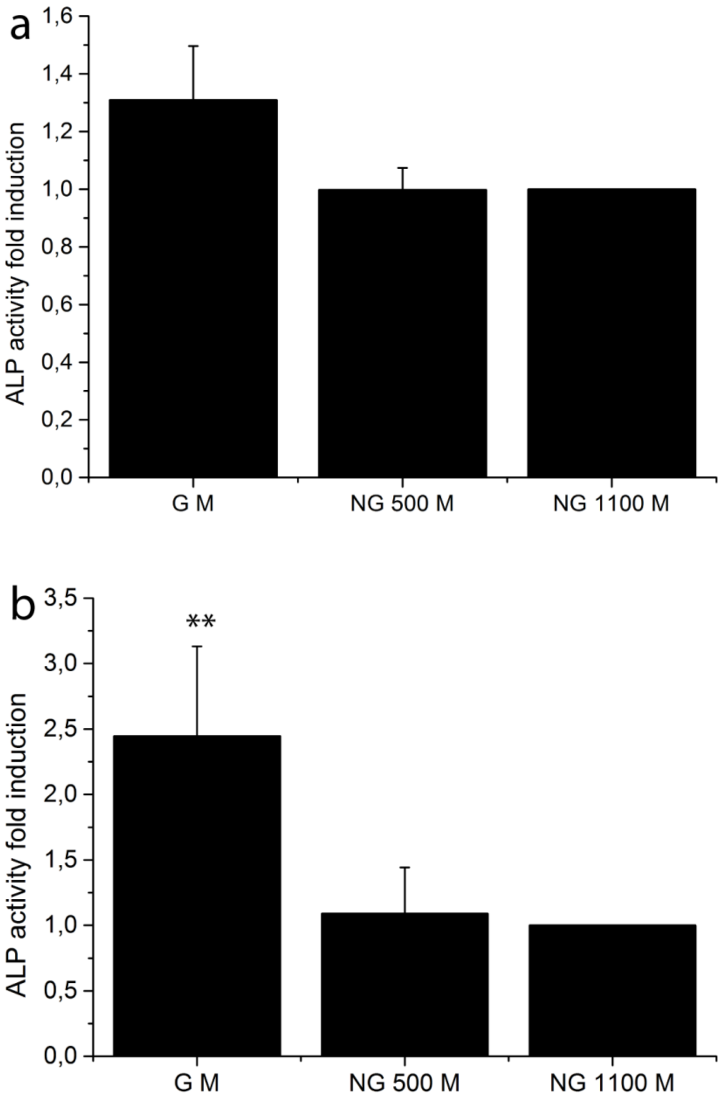

Figure S3. ALP activity of D1 (a) and D2 (b) expressed as fold induction normalized by the ALP activity levels of NG1100. In D1 the ALP activity was increased by 25% and significantly increased by 2.5 times in D2. A normalization by NG500 gave the same fold induction. ** statistical significance $p<0.01$.

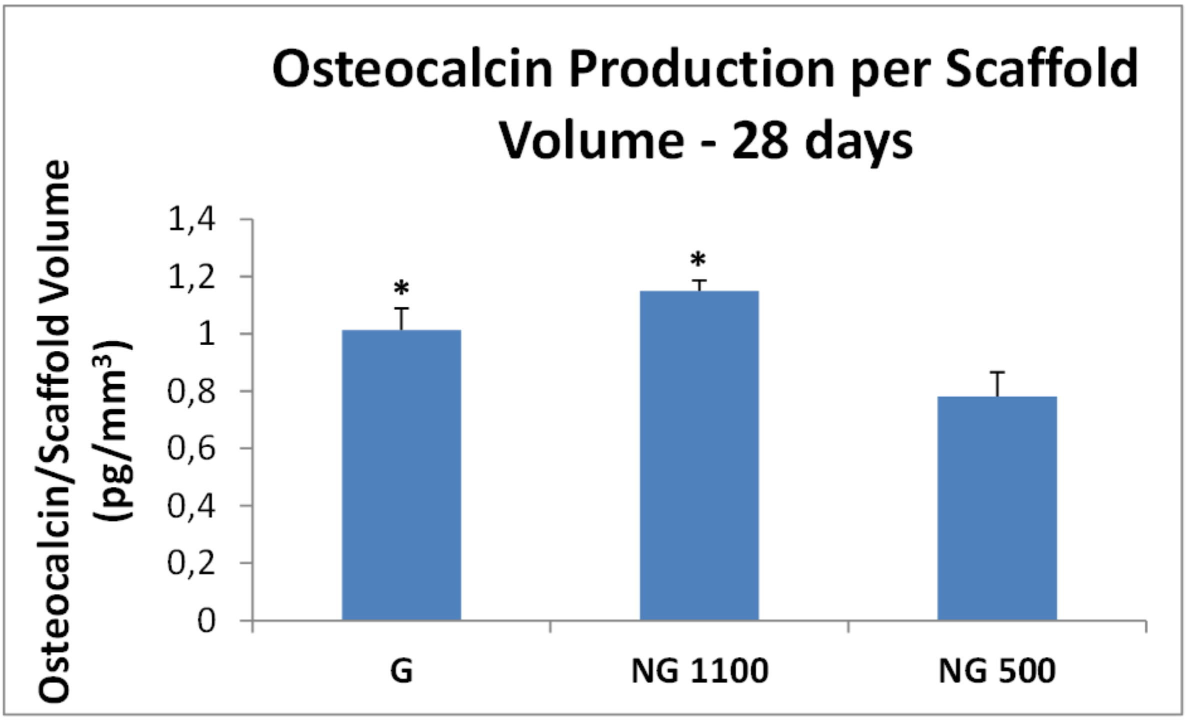

Figure S4. Osteocalcin production measured by ELISA assay on G, NG 1100 and NG 500 scaffolds show significantly higher amounts of osteocalcin in G and NG 1100 scaffolds, compared to NG 500 scaffolds. * indicates statistical significance with $p < 0.05$.

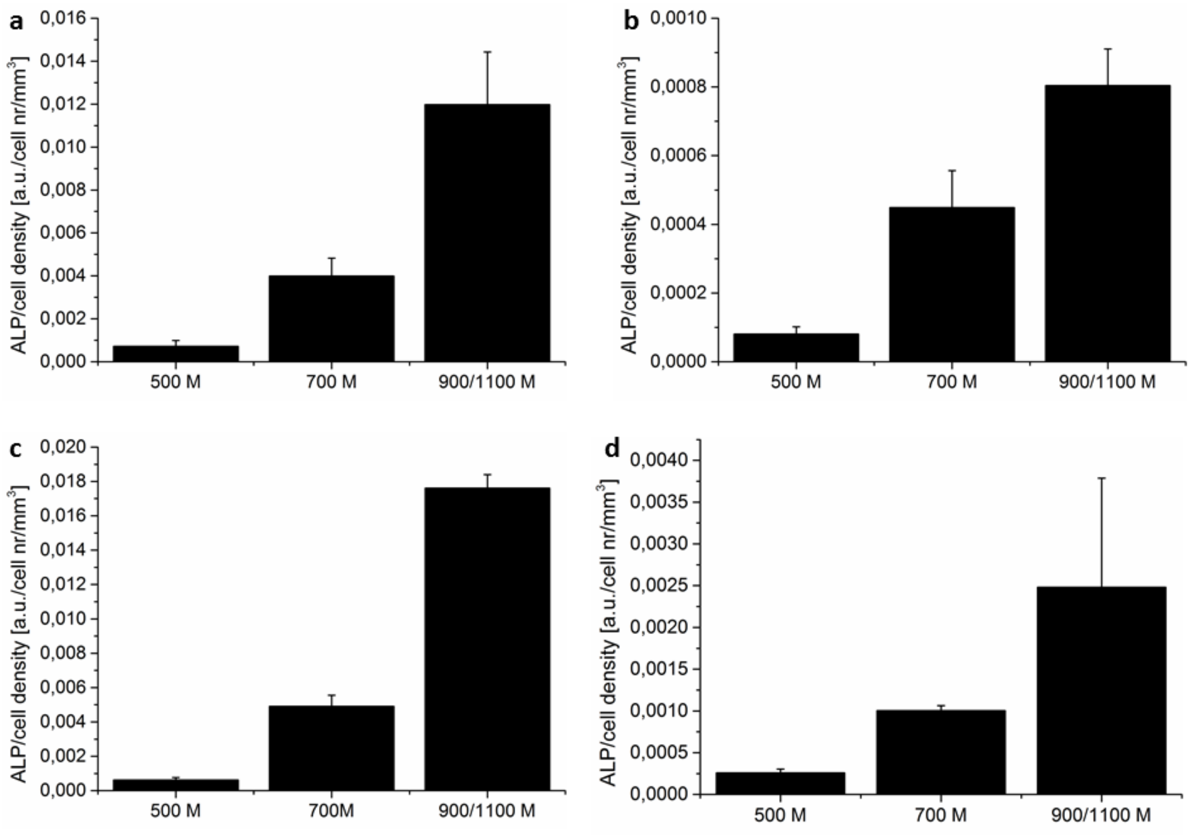

Figure S5. Partition analysis of ALP activity of D1 (a, c) and D2 (b, d) in 300PEOT55PBT (a, b) and PCL (c, d) gradient scaffolds normalized by cell density. ** shows statistical significance $p<0.01$.

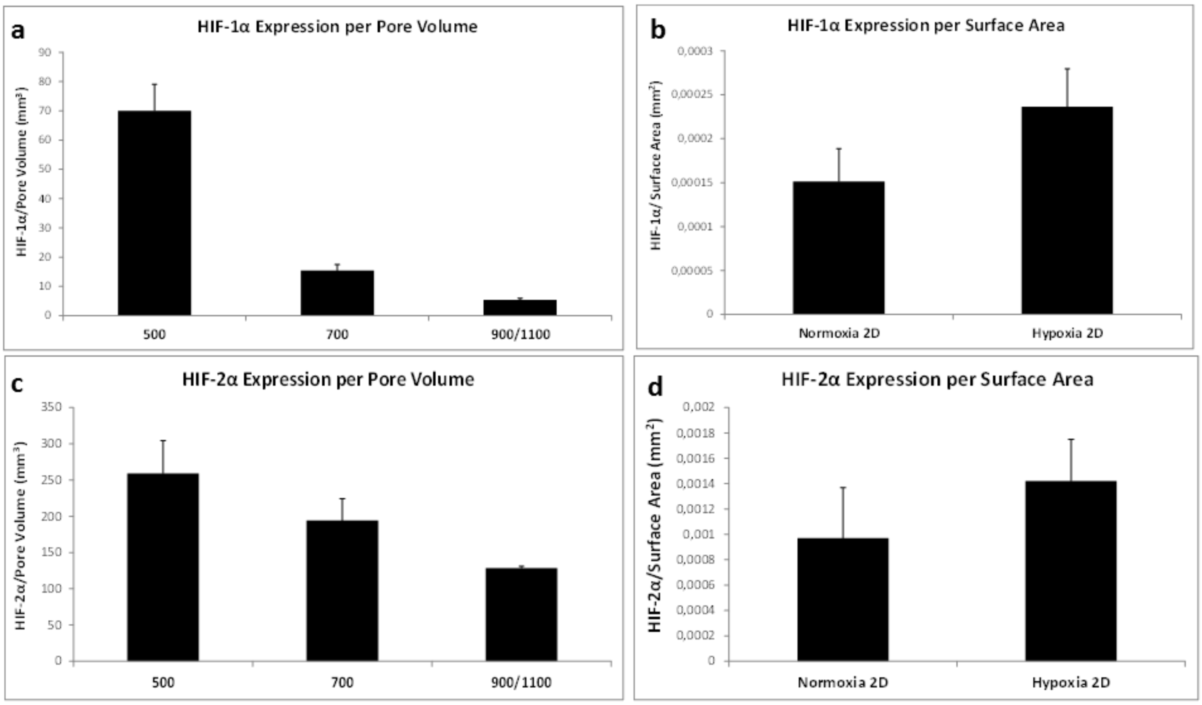

Figure S6. HIF-1α (a) and HIF-2α (c) expression in the different regions of gradients scaffolds, showing a decrease of HIF factors with increasing the pore size. (b, d) contro experiments in 2D cell culture plates in normoxic and hypoxic conditions.
